# Willingness to Get the COVID-19 Vaccine among Residents of Slum Settlements

**DOI:** 10.3390/vaccines9090951

**Published:** 2021-08-26

**Authors:** Juan P. Aguilar Ticona, Nivison Nery, Renato Victoriano, Mariam O. Fofana, Guilherme S. Ribeiro, Emanuele Giorgi, Mitermayer G. Reis, Albert I. Ko, Federico Costa

**Affiliations:** 1 Instituto de Saúde Coletiva, Universidade Federal da Bahia, Rua Basílio da Gama S/N, Canela, Salvador 40110-040, Brazil; juanpat@ufba.br (J.P.A.T.); nivison.nery@fiocruz.br (N.N.J.); 2 Instituto Gonçalo Moniz, Fundação Oswaldo Cruz, Ministério da Saúde, Rua Waldemar Falcão, 121, Candeal, Salvador 40296-710, Brazil; renato.victoriano@fiocruz.br (R.V.); guilherme.ribeiro@fiocruz.br (G.S.R.); miter@bahia.fiocruz.br (M.G.R.); 3 Department of Epidemiology of Microbial Diseases, Yale School of Public Health, Yale University, 60 College Street, P.O. Box 208034, New Haven, CT 06520-8034, USA; mariam.fofana@yale.edu; 4 Faculdade de Medicina da Bahia, Universidade Federal da Bahia, Praça XV de Novembro, s/n, Largo do Terreiro de Jesus, Salvador 40026-010, Brazil; 5 School of Health and Medicine, Lancaster University, Bailrigg, Lancaster LAI 4YB, UK; e.giorgi@lancaster.ac.uk

**Keywords:** COVID-19, vaccine, vaccine hesitancy

## Abstract

Slum residents are more vulnerable to COVID-19 infection. Without a specific treatment, vaccination became the main strategy against COVID-19. In this study, we determined the rate and factors associated with the willingness to get vaccinated against COVID-19 among slum residents and their main reasons associated with the vaccine intention. The study was conducted in Pau da Lima, a slum community in Salvador Brazil. In total, 985 residents were interviewed. Among them 66.0% (650/985) were willing to get vaccinated, 26.1% (257/985) were hesitant to take the vaccine and 7.9% (78/285) were not sure. The main reasons cited for vaccine hesitancy or being unsure were concerns about vaccine efficacy and potential side effects. In contrast, the main reasons cited for wanting the vaccine were the high incidence of COVID-19 cases and participants’ self-perception of their own health history. Multivariate analysis identified that COVID-19 vaccine hesitancy was associated with younger age and low social capital, summarized as low perceived importance of vaccination to protect one’s family, friends and community. Slum residents have been less willing to vaccinate than the general population. Social capital presents a critical opportunity in the design of communication campaigns to increase COVID-19 vaccine acceptance in slum settings.

## 1. Introduction

About 30% of the world’s population lives in slum settlements [1]. Slum residents suffer from higher rates of COVID-19 infection, hospitalization, and mortality than their wealthy counterparts [2]. Recent research has identified that more than 50% of residents in these communities were exposed to the SARS-CoV-2 [2,3]. Moreover, COVID-19 has had a large economic impact due to rising unemployment rates, which hamper adhesion to preventive measures (such as lockdown and social distancing) [4]. Vaccination campaigns are the main strategy to decrease this COVID-19 burden. However, the lack of information regarding COVID-19 vaccine acceptance [5] in urban slum communities may limit the success of vaccination campaigns and impact on COVID-19 vaccination coverage.

Vaccine hesitancy is a public health issue that has increased in recent years [6,7,8]. Mainly it is associated with doubts and concerns regarding vaccination, and also due to the risk perception reduction of vaccine-preventable diseases sequels [6]. These phenomena result in the recent outbreaks as measles [9,10], and pertussis [11]. In the COVID-19 context, vaccine hesitancy represents a challenge to disease control due to the fast development and the misinformation that were applied through social media [8,12]. Moreover, a previous review identified an increasing trend of low or no intention to vaccinate against COVID-19 ratio in common population during 2020 [13] highlighting the importance of monitoring the vaccine hesitancy. As consequence, the aim of this study was to determine the frequency and factors associated with the willingness to get the COVID-19 vaccine among slum residents.

## 2. Methods

Based on an ongoing cohort study, we recruited non-pregnant participants aged 18 years or older in Pau da Lima, a slum community in Salvador, Brazil (13°32′53.47″ S; 38°43′51.10″ W) [14]. It was estimated that the population of Salvador in 2020 was 2.9 million and approximately 3% of population lived in the Pau da Lima study surveillance site (IBGE) [15]. The study area is conforming for three-valley site with an area of 0.46 km^2^. Previous study census identified 3689 households within the area. This urban slum notably has significant socioeconomic determinants, such as low median income (55% have a per capita household income of <USD 2.5 per day), the presence of illegal settlements, and substandard sanitation [16].

Slum residents were asked to participate by completing a structured questionnaire between 16 November 2020 and 28 February 2021. The questionnaire was divided in the following sections: (A) demographic characteristics, including age (in years), sex, ethnicity, schooling, marital status, employment, income, and comorbidities. (B) COVID-19 diagnoses and exposures, including the presence of symptoms associated with COVID-19, previous COVID-19 suspicions, COVID-19 laboratorial test results, information about household members and the COVID-19 exposure. (C) If participants received influenza vaccine in 2020. (D) Finally, attitudes related to COVID-19 risk and vaccination, including, willingness to get the COVID-19 vaccine, the main reason for vaccine acceptance or hesitancy, the risk perception, and the importance of vaccination to protect their families, friends and community. Willingness to get the COVID-19 vaccine was assessed with the question, “If there were a safe and effective vaccine to prevent COVID-19, would you be interested in getting the vaccine?” Response options included, “Yes”, “No”, or “Not sure”.

Data were summarized using descriptive statistics. The Chi-squared test and *t*-test were used for categorical and numeric variables, to assess differences between participants who answered “Yes” and those who answered “No” to the willingness to get the COVID-19 vaccine question. We also assessed differences between participants who answered “Yes” and those who answered “Not sure”. A *p*-value of <0.05 was considered to be statistically significant. We performed a multivariable analysis using multinomial logistical regression to determine factors associated with the willingness to get the COVID-19 vaccine. To measure associations, we used odds ratio (OR) and 95% confidence intervals (CIs). Model specification was performed by the forward-selection method.

## 3. Results

**Figure 1:**
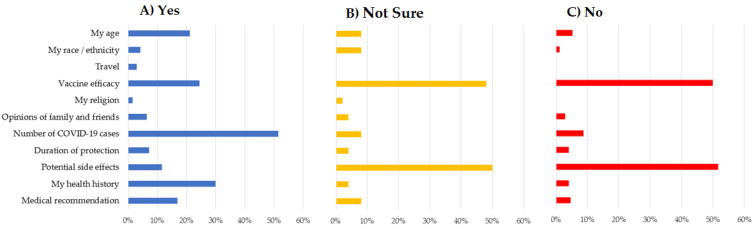
Reasons provided by participants regarding willingness to receive a COVID-19 vaccine. (**A**) Among participants who demonstrated willingness to get vaccinated; (**B**) among participants who were unsure about vaccination; (**C**) among participants who hesitant to take the COVID-19 vaccine.

We recruited 985 residents, with a mean age of 39 years (SD 14), of whom 394 40% (390/985) were male and 54% (531/985) self-identified as black. Overall, 66.0% (650/985) of residents were willing to get vaccinated (answered “Yes”), 26.1% (257/985) hesitant (answered “No”) to take the vaccine and 7.9% (78/285) were not sure (Table 1). The main reasons cited for vaccine hesitancy or being unsure were concerns about vaccine efficacy and potential side effects. On the other hand, the main reasons cited for wanting the vaccine were the high incidence of COVID-19 cases and participants’ self-perception of their own health history (Figure 1).

We identified several factors associated with vaccine refusal or uncertainty, including demographic and socioeconomic characteristics, and COVID-19 exposures. Factors associated with COVID-19 vaccine hesitancy in the univariable analysis were younger age and lower per capita daily income. On the other hand, participants who were willing to get vaccinated were more likely to have an underlying medical condition, have previously undergone COVID-19 molecular testing, have received influenza vaccine in 2020, and be currently employed in the informal sector (Table 1). 

Moreover, attitudes associated with vaccine acceptance included the perception of being at high risk for COVID-19, thinking that it is a serious disease, and the belief that the vaccine would not only protect those who would receive it but also their families, friends and community (Table 2). Finally, among a sub-sample of 402 parents, 67% (270/402) indicated that, if the vaccine were available and safe for children, they would vaccinate their children, whereas 18% (73/402) would reject the vaccine for their children and 15% (59/409) were unsure. This attitude was positively associated with the parents’ intention to vaccinate themselves (Table 2).

**Figure 2:**
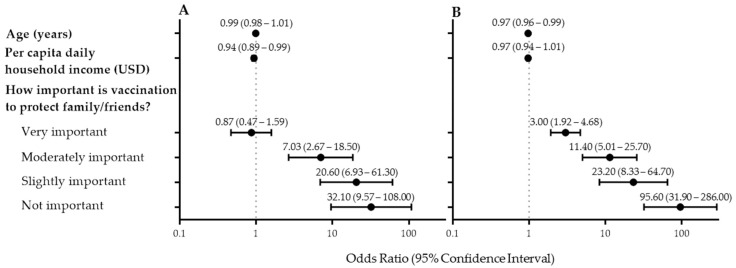
Multinomial regression model of factors associated with COVID-19 vaccine hesitancy. (**A**) Not Sure vs. Yes (**B**) No vs. Yes.

In a multinomial logistic regression model, COVID-19 vaccine hesitancy was associated with younger age and low perceived importance of vaccination to protect one’s family and friends (Figure 2 and Table S1). The perceived importance of being vaccinated to protect family and friends was highly concordant with the perceived importance of being vaccinated to protect the community (Cohen’s kappa = 0.94). Multivariable models including the perceived importance of getting vaccinated to protect family or the perceived importance of getting vaccinated to protect the community had similar fits.

## 4. Discussion

In our survey of residents of an urban slum, we found that 66.0% would be willing to accept a COVID-19 vaccine. This is lower than previously reported in an online survey, that estimated 85.4% and 75.1% willingness to get vaccinated in Brazil and globally, respectively [17]. Differences in acceptance between this and prior studies may be due to underrepresentation of slum dwellers in vaccine surveys, especially those that require participants to have access to computers or mobile devices with an internet connection. These differences may also be attributable to variation in demographic and socioeconomic factors associated with vaccine acceptance, as prior studies suggest that age and wealth are associated with increased vaccine acceptance [13,17]. 

Believing that the vaccine would not only protect those who receive it but also their families and friends was positively associated with the willingness to receive the vaccine. This finding suggests that social capital may have an important role in COVID-19 vaccination campaigns in the slum setting since communities with a high social capital have better adherence to preventive measures [18]. Moreover, we found that residents are also willing to get the COVID-19 vaccine to protect other residents of their community. Similar findings regarding social capital among individuals without a closely personal connection have also been reported in studies focusing on COVID-19 preventive measures not related to vaccination [18,19].

Finally, concerns regarding vaccine efficacy and potential side effects featured prominently among the drivers of willingness to get vaccinated. This indicates that survey participants are making decisions based on their understanding of available information, and that attitudes towards vaccination may respond favorably to education on the benefits and safety of vaccination. On the other hand, exposure to misinformation may seriously hamper vaccination efforts [20]. Governments need to provide information about COVID-19 vaccination using contemporary communication channels as social media. Uncertainties around the COVID-19 and the vaccination can be an opportunity to spread misinformation by social media, fast and to a large number of people. Social media also represent an opportunity to solve the population uncertainties. It was demonstrated in some studies that use it not only to monitor concerns regarding the COVID-19 epidemic [21]. However, also using health education and digital communication as strategy against the COVID-19 vaccine misinformation [22]. 

While this study was restricted to a specific community, our community-based approach provides a critical view of the general COVID-19 vaccine acceptance in slum communities. Addressing the concerns about vaccine effectiveness and safety in low-income sub-groups and younger adult population groups will be key in increasing vaccine uptake. Our findings suggest urban slum residents have lower willingness to vaccinate than the general population and that social capital—the perceived benefit of protecting family and community—presents a critical opportunity in the design of communication campaigns to increase COVID-19 vaccine acceptance in slum settings.

## Figures and Tables

**Table 1:** Participants’ demographic characteristics and COVID-19 diagnoses and exposures among Pau da Lima residents and willingness to receive COVID-19 vaccination.

Characteristic	No. of Responses	Survey Sample ^1^ (*N* = 985)	Willingness to Receive a COVID-19 Vaccine ^2^			*p*-Value ^3^ Yes vs. Not Sure	*p*-Value ^3^ Yes vs. No
			Yes (*n* = 650)	Not Sure (*n*= 78)	No (*n* = 257)		
		Mean (SD) or *n* (%)					
Demographic characteristics							
Age in years	985	39 (14)	40 (15)	38 (15)	35 (13)	0.4	<0.001
Gender						0.33	0.43
Male	985	394 (40)	258 (65)	26 (6.6)	110 (28)		
Female	985	591 (60)	392 (66)	52 (8.8)	147 (25)		
Ethnicity						0.057	0.47
Black	985	531 (54)	354 (67)	37 (7.0)	140 (26)		
Brown	985	396 (40)	261 (66)	34 (8.6)	101 (26)		
White	985	51 (5.2)	33 (65)	5 (9.8)	13 (25)		
Others	985	7 (0.7)	2 (29)	2 (29)	3 (43)		
Schooling						0.39	0.63
0–6 years	985	342 (35)	226 (66)	31 (9.1)	85 (25)		
≥7 years	985	643 (65)	424 (66)	47 (7.3)	172 (27)		
Married or stable union						0.92	0.44
Yes	985	358 (36)	242 (68)	28 (7.8)	88 (25)		
No	985	627 (64)	408 (65)	50 (8.0)	169 (27)		
Employment						0.21	0.018
Formal	985	309 (31)	190 (61)	27 (8.7)	92 (30)		
Informal	985	182 (18)	137 (75)	10 (5.5)	35 (19)		
Unemployed	985	494 (50)	323 (65)	41 (8.3)	130 (26)		
Per capita daily household income (USD)	985	5.2 (5.5)	5.6 (5.8)	4.1 (4.6)	4.5 (5.0)	0.011	0.007
Lost employment during pandemic						0.008	0.62
Yes	952	400 (42)	278 (70)	19 (4.8)	103 (26)		
No	952	552 (58)	356 (64)	52 (9.4)	144 (26)		
Underlying medical condition ^4^						0.38	0.01
Yes	985	216 (22)	159 (74)	15 (6.9)	42 (19)		
Received influenza vaccine in 2020						0.004	<0.001
Yes	665	253 (38)	186 (74)	18 (7.1)	49 (19)		
No	665	412 (62)	235 (57)	53 (13)	124 (30)		
No	985	769 (78)	491 (64)	63 (8.2)	215 (28)		
COVID-19 diagnoses and exposures							
Episode of COVID-19 symptoms						0.14	0.57
Yes	985	110 (11)	73 (66)	4 (3.6)	33 (30)		
No	985	875 (89)	577 (66)	74 (8.5)	224 (26)		
Clinical suspicion of COVID-19						>0.99	0.55
Yes	982	31 (3.2)	22 (71)	3 (9.7)	6 (19)		
No	982	951 (97)	627 (66)	75 (7.9)	249 (26)		
Tested for COVID-19						0.046	0.068
Yes	985	149 (15)	112 (75)	6 (4.0)	31 (21)		
No	985	836 (85)	538 (64)	72 (8.6)	226 (27)		
Household member with suspected COVID-19						0.19	>0.99
Yes	619	76 (12)	53 (70)	3 (3.9)	20 (26)		
No	619	543 (88)	358 (66)	50 (9.2)	135 (25)		
Household member with confirmed COVID-19						>0.99	0.65
Yes	615	18 (2.9)	11 (61)	1 (5.6)	6 (33)		
No	615	597 (97)	396 (66)	52 (8.7)	149 (25)		
Received molecular testing						0.19	0.008
Yes	983	70 (7.1)	58 (83)	3 (4.3)	9 (13)		
No	983	913 (93)	591 (65)	75 (8.2)	247 (27)		
Positive molecular test result among tested						>0.99	0.92
Yes	70	13 (19)	11 (85)	1 (7.7)	1 (7.7)		
No	70	57 (81)	47 (82)	2 (3.5)	8 (14)		
Received serological testing						0.12	0.92
Yes	980	91 (9.3)	64 (70)	3 (3.3)	24 (26)		
No	980	889 (91)	583 (66)	75 (8.4)	231 (26)		
Positive serologic test result among tested						0.11	>0.99
Yes	91	14 (15)	9 (64)	2 (14)	3 (21)		
No	91	77 (85)	55 (71)	1 (1.3)	21 (27)		

^1^ Survey sample column was summarized using column percentages; ^2^ Willingness to take COVID-19 vaccine columns were summarized using row percentages; **^3^** *t*-test or Pearson’s Chi-squared test; ^4^ Hypertension, diabetes and cancer.

**Table 2:** Attitudes related to COVID-19 risk and vaccination among Pau da Lima residents and the willingness to receive COVID-19 vaccination.

Characteristic	No. of Responses	Survey Sample ^1^ (*N* = 985)	Willingness to Receive a COVID-19 Vaccine ^2^			*p*-Value ^3^ Yes vs. Not Sure	*p*-Value ^3^ Yes vs. No
			Yes (*n* = 650)	Not Sure (*n* = 78)	No (*n* = 257)		
		Mean (SD) or *n* (%)					
How likely are you to get COVID-19?						0.42	0.021
Very likely	985	240 (24)	169 (70)	16 (6.7)	55 (23)		
Moderately likely	985	225 (23)	154 (68)	18 (8.0)	53 (24)		
Slightly likely	985	222 (23)	141 (64)	23 (10)	58 (26)		
Not likely	985	164 (17)	92 (56)	13 (7.9)	59 (36)		
Do not know	985	134 (14)	94 (70)	8 (6.0)	32 (24)		
How severe would your condition be if you contracted COVID-19?						0.041	0.011
Very severe	985	242 (25)	169 (70)	9 (3.7)	64 (26)		
Moderately severe	985	155 (16)	102 (66)	18 (12)	35 (23)		
Slightly severe	985	216 (22)	142 (66)	22 (10)	52 (24)		
Not severe	985	152 (15)	83 (55)	12 (7.9)	57 (38)		
Do not know	985	220 (22)	154 (70)	17 (7.7)	49 (22)		
How important is vaccination to protect family/friends?						<0.001	<0.001
Extremely important	821	311 (38)	256 (82)	24 (7.7)	31 (10.0)		
Very important	821	374 (46)	253 (68)	22 (5.9)	99 (26)		
Moderately important	821	41 (5.0)	12 (29)	9 (22)	20 (49)		
Slightly important	821	33 (4.0)	6 (18)	11 (33)	16 (48)		
Not important	821	62 (7.6)	4 (6.5)	12 (19)	46 (74)		
How important is vaccination to protect the health of your community?						<0.001	<0.001
Extremely important	821	295 (36)	244 (83)	22 (7.5)	29 (9.8)		
Very important	821	364 (44)	252 (69)	21 (5.8)	91 (25)		
Moderately important	821	46 (5.6)	15 (33)	9 (20)	22 (48)		
Slightly important	821	48 (5.8)	10 (21)	15 (31)	23 (48)		
Not important	821	68 (8.3)	10 (15)	11 (16)	47 (69)		
Would you vaccinate your children if safe and effective? ^4^		402				<0.001	<0.001
Yes	402	270 (67)	224 (83)	20 (7.4)	26 (9.6)		
No	402	73 (18)	12 (16)	4 (5.5)	57 (78)		
Do not know	402	59 (15)	17 (29)	22 (37)	20 (34)		

^1^ Survey sample column was summarized using column percentages; ^2^ Willingness to take COVID-19 vaccine columns were summarized using row percentages; **^3^** *t*-test or Pearson’s Chi-squared test. ^4^ Information available only for parents; the variation in number of responses between variables reflects the availability of specific information.

## Data Availability

The data for this article will be shared on reasonable request to the corresponding author.

## Supplementary Materials

The following are available online at https://www.mdpi.com/article/10.3390/vaccines9090951/s1, Table S1: Multinomial regression model of factors associated with COVID-19 vaccine hesitancy.
