# Supplementary material for: Willingness to Get the COVID-19 Vaccine among Residents of Slum Settlements
Source: Vaccines (Basel). 2021 Aug 26;9(9):951. doi: 10.3390/vaccines9090951 (PMC8472908; doi:10.3390/vaccines9090951)
Supplement: Supplementary file 1 [file vaccines-09-00951-s001.zip › vaccines-1327520-supplementary.pdf]

Table S1. Multinomial regression model of factors associated with COVID-19 vaccine hesitancy

Characteristic	Univariable		Multivariable	
	Yes vs Not Sure	Yes vs No	Not Sure vs Yes	No vs Yes
	OR (95% CI)	OR (95% CI)	OR (95% CI)	OR (95% CI)
Age (years)	0.99 (0.98 – 1.01)	0.98 (0.97 – 0.99)	0.99 (0.98 – 1.01)	0.97 (0.96 – 0.99)
Schooling				
0 to 6	1.24 (0.76 – 1.99)	0.93 (0.68 – 1.26)		
≥ 7	—	—		
Employment				
Formal	—	—		
Informal	0.51 (0.24 – 1.10)	0.53 (0.34 – 0.82)		
Unemployed	0.89 (0.53 – 1.51)	0.83 (0.60 – 1.15)		
Per capita daily household income (USD)	0.95 (0.90 – 0.99)	0.96 (0.94 – 0.99)	0.94 (0.89 – 0.99)	0.97 (0.94 – 1.01)
Underlying medical condition [1]	0.74 (0.39 – 1.29)	0.6 (0.41 – 0.87)		
Have you ever been tested to see if you have COVID-19?	0.4 (0.15 – 0.87)	0.66 (0.42 – 1.00)		
Received molecular testing	0.41 (0.10 – 1.14)	0.37 (0.17 – 0.72)		
Receive the influenza vaccine in 2020?	0.43 (0.24 – 0.74)	0.5 (0.34 – 0.73)		
How likely are you to get the the COVID-19?				
Very probable	—	—		
Moderately probable	1.23 (0.61 – 2.53)	1.06 (0.68 – 1.64)		
Slightly probable	1.72 (0.88 – 3.44)	1.26 (0.82 – 1.95)		
Not probable	1.49 (0.68 – 3.24)	1.97 (1.26 – 3.09)		
Don't know	0.9 (0.35 – 2.12)	1.05 (0.63 – 1.72)		
How severe would the COVID-19 be?				
Very severe	—	—		
Moderately severe	3.31 (1.47 – 7.99)	0.91 (0.56 – 1.46)		
Slightly severe	2.91 (1.34 – 6.85)	0.97 (0.63 – 1.48)		
Not severe	2.71 (1.11 – 6.90)	1.81 (1.16 – 2.83)		
Don't know	2.07 (0.92 – 4.99)	0.84 (0.54 – 1.29)		
How important is vaccination to protect family/friends?				
Extremely important	—	—	—	—
Very important	0.93 (0.50 – 1.70)	3.23 (2.11 – 5.08)	0.87 (0.47 – 1.59)	3 (1.92 – 4.68)
Moderately important	8 (3.00 – 20.9)	13.8 (6.24 – 31.7)	7.03 (2.67 – 18.5)	11.4 (5.01 – 25.7)
Slightly important	19.6 (6.84 – 61.3)	22 (8.40 – 65.3)	20.6 (6.93 – 61.3)	23.2 (8.33 – 64.7)

Not important	32 (10.3 – 122)	95 (35.7 – 331)	32.1 (9.57 – 108)	95.6 (31.9 – 286)
How important is vaccination to protect the health of your community?				
Extremely important	—	—		
Very important	0.92 (0.49 – 1.73)	3.04 (1.95 – 4.85)		
Moderately important	6.65 (2.54 – 16.8)	12.3 (5.83 – 26.9)		
Slightly important	16.6 (6.79 – 42.7)	19.4 (8.61 – 46.5)		
Not important	12.2 (4.67 – 32.5)	39.5 (18.8 – 90.9)		

OR = Odds Ratio – CI = Confidence Interval

[1] Hypertension, diabetes and cancer
